# Repeated exposure reduces the response to impulsive noise in European seabass

**DOI:** 10.1111/gcb.13352

**Published:** 2016-06-10

**Authors:** Andrew N. Radford, Laurie Lèbre, Gilles Lecaillon, Sophie L. Nedelec, Stephen D. Simpson

**Affiliations:** ^1^ School of Biological SciencesUniversity of BristolLife Sciences Building24 Tyndall AvenueBristolBS8 1TQUK; ^2^ Écocéan33 rue Chaptal34 000MontpellierFrance; ^3^ BiosciencesCollege of Life and Environmental SciencesUniversity of ExeterStocker RoadExeterEX4 4QDUK

**Keywords:** anthropogenic noise, *Dicentrarchus labrax*, European seabass, growth, habituation, hearing threshold, pollution, stress, tolerance, ventilation rate

## Abstract

Human activities have changed the acoustic environment of many terrestrial and aquatic ecosystems around the globe. Mounting evidence indicates that the resulting anthropogenic noise can impact the behaviour and physiology of at least some species in a range of taxa. However, the majority of experimental studies have considered only immediate responses to single, relatively short‐term noise events. Repeated exposure to noise could lead to a heightened or lessened response. Here, we conduct two long‐term (12 week), laboratory‐based exposure experiments with European seabass (*Dicentrarchus labrax*) to examine how an initial impact of different sound types potentially changes over time. Naïve fish showed elevated ventilation rates, indicating heightened stress, in response to impulsive additional noise (playbacks of recordings of pile‐driving and seismic surveys), but not to a more continuous additional noise source (playbacks of recordings of ship passes). However, fish exposed to playbacks of pile‐driving or seismic noise for 12 weeks no longer responded with an elevated ventilation rate to the same noise type. Fish exposed long‐term to playback of pile‐driving noise also no longer responded to short‐term playback of seismic noise. The lessened response after repeated exposure, likely driven by increased tolerance or a change in hearing threshold, helps explain why fish that experienced 12 weeks of impulsive noise showed no differences in stress, growth or mortality compared to those reared with exposure to ambient‐noise playback. Considering how responses to anthropogenic noise change with repeated exposure is important both when assessing likely fitness consequences and the need for mitigation measures.

## Introduction

Human activities, such as energy production, resource extraction, urban development and transportation, have changed the acoustic environment across the globe (Barber *et al*., 2009; Slabbekoorn *et al*., 2010; Normandeau Associates, Inc., 2012). In addition to increasing the amount of acoustic energy, these activities often generate sounds that are different from those arising from natural sources (Hildebrand, 2009; Normandeau Associates, Inc., 2012). Many recent studies have shown that the resulting anthropogenic noise can have an impact on the behaviour and physiology of at least some organisms, as well as on community structure and ecosystem function (Barber *et al*., 2009; Slabbekoorn *et al*., 2010; Morley *et al*., 2014; Shannon *et al*., 2016). However, the majority of experimental work to date has measured responses only once and/or to single, relatively short‐term noise exposures (e.g. Halfwerk & Slabbekoorn, 2009; McLaughlin & Kunc, 2013; Simpson *et al*., 2015, 2016). While that research has produced undoubtedly important knowledge, experimental investigation of the possibility that responses might change with repeated exposure (Bejder *et al*., 2009; Radford *et al*., 2015) is crucial both for a full understanding of the fitness consequences of noise exposure and for an accurate assessment of the need for mitigation measures.

Response moderation to repeated stimulus exposure can potentially result from a change in individual tolerance levels (Nisbet, 2000) or, in the case of noise stimuli, a shift in hearing threshold (Popper & Hastings, 2009). An increased responsiveness over time could arise through sensitization, when animals become less tolerant as they learn that the stimulus has significant consequences for them (Richardson *et al*., 1995). Higher levels of human disturbance have been shown to result in heightened responses, such as increased levels of stress hormones, in a variety of species (e.g. Ellenberg *et al*., 2007; Strasser & Heath, 2013; Menard *et al*., 2014). A decreased responsiveness over time could also arise through a change in tolerance, through habituation – persistent waning of responsiveness if repeated stimulation is not followed by reinforcement (Thorpe, 1963). Reduced behavioural and physiological responses to continued human disturbance have been described in a number of studies (e.g. Ellenberg *et al*., 2009; Ensminger & Westneat, 2012; Viblanc *et al*., 2012). A decreased responsiveness over time to noise stimuli could alternatively arise from a shift in hearing threshold; some sources of anthropogenic noise have been shown to cause temporary threshold shifts (transient reductions in hearing sensitivity) in some, but not all, tested fish species (Scholik & Yan, 2001; Popper *et al*., 2005, 2007; Wysocki *et al*., 2007). To establish whether there is a change in responsiveness to a particular stimulus requires repeated sampling of the same cohort of individuals across time (Nisbet, 2000; Bejder *et al*., 2009), something which has only rarely been attempted with respect to anthropogenic noise (Halfwerk *et al*., 2012; Wale *et al*., 2013a; Nedelec *et al*., 2015, in press).

The impact of anthropogenic noise is likely to be affected not only by its level, but also by the characteristics of the sound (Slabbekoorn *et al*., 2010; Gill *et al*., 2015; Nedelec *et al*., 2015); man‐made noise sources differ greatly in such aspects as frequency range, amplitude fluctuation and temporal structure (Hildebrand, 2009; Gill *et al*., 2015). For instance, pile‐driving and seismic airguns produce intermittent, impulsive sounds, whereas ships produce intermittent but not impulsive sounds, and wind turbines produce more continuous sounds. Most experimental studies so far have focused on the effect of a single sound type, but recent work has demonstrated that fish behavioural responses and recovery differ depending on the intermittency of short‐term (30 min) sound exposures (Neo *et al*., 2014). Whether and how responses change with repeated exposure to different sound types, and the possibility of generalization (changed response to more than just the source to which an organism has been exposed), are important issues for managers and policymakers.

Here, we report the results from laboratory‐based, long‐term exposure experiments on juvenile European seabass (*Dicentrarchus labrax*), which examined the immediate and changing effect of various types of noise. Caution is needed when extrapolating from captivity to the wild, as important behavioural and acoustic differences exist (e.g. Rogers, 2015; Slabbekoorn, 2015). But, laboratory studies allow careful control of potential confounding factors, detailed data collection and guaranteed noise exposure at required levels over extended periods of time (Slabbekoorn, 2015). Captive studies therefore provide a valuable stepping stone in the study of environmental stressors (Dixson *et al*., 2010; Scott & Johnson, 2012), including anthropogenic noise (Wale *et al*., 2013a,b; Nedelec *et al*., 2015; Simpson *et al*., 2015).

All fish species that have been studied are capable of hearing, with many demonstrably using environmental sounds and both conspecific and heterospecific acoustic communications to inform behavioural decisions (Bone & Moore, 2008; Radford *et al*., 2014). As such, fishes are potentially vulnerable to anthropogenic noise, and there is increasing evidence that at least some species are detrimentally affected in terms of their behaviour (e.g. Picciulin *et al*., 2010; Bruintjes & Radford, 2013; Simpson *et al*., 2015, 2016) and physiology (e.g. Wysocki *et al*., 2006; Anderson *et al*., 2011; Simpson *et al*., 2015, 2016). As fish are socio‐economically important, yet many species are vulnerable to anthropogenic pressures such as overfishing, ocean acidification and global warming (Harley *et al*., 2006; Kroeker *et al*., 2010; Simpson *et al*., 2011), they are a key taxon to consider with respect to anthropogenic noise. Fish studies to date have mostly examined short‐term impacts of additional noise; mixed results have arisen from the limited number of longer‐term experiments (see Wysocki *et al*., 2007; Davidson *et al*., 2009; Anderson *et al*., 2011; Bruintjes & Radford, 2014; Nedelec *et al*., 2015, in press) and there has been little investigation of changing levels of response with repeated exposure.

European seabass are commercially important and there is recent evidence that their physiology is affected by short‐term playback of pile‐driving noise (Bruintjes *et al*., 2016), as well as actual pile‐driving events (Debusschere *et al*., 2016). In the current study, we first tested the effect of short‐term noise exposure on naïve juvenile fish (those that had received no previous noise playbacks). We compared responses to playbacks of impulsive sound types (recordings of pile‐driving and seismic surveys) and a more continuous sound type (recordings of ship passes), using playback of recordings of ambient coastal noise as a control. Recordings of real‐world noise sources were used as exemplars of sound types with different acoustic characteristics to test general principles relating to a potential change in response with repeated exposure, rather than to provide information about absolute responses to those particular noise sources. We then exposed cohorts of fish to 12 weeks of each sound type, before investigating whether the initial impacts of short‐term exposure were still apparent or whether there had been changes in response. Having demonstrated decreased levels of response, we examined the implications of long‐term exposure to different sound types for stress, growth and mortality.

## Material and methods

### Ethics

This research adhered to the Association for the Study of Animal Behaviour/Animal Behavior Society Guidelines for the Use of Animals in Research, the legal requirements of the country (France) in which the work was carried out and all institutional guidelines (University of Bristol Animal Services Ethical Committee approval: UB/10/034). Fish showed no signs of pain, suffering, distress or lasting harm during the study; animals were killed by Schedule 1 methods at the end of the experiments.

### Study species and holding conditions

Postlarval seabass, captive bred from stock that had been wild‐caught >10 years previously, were obtained from Les Poissons du Soleil, Balaruc‐les‐Bains, France, approximately 1 month posthatching. Fish were transferred to the experimental laboratory at Centre de Recherche sur les Écosystèmes Marins (CREM), Le Barcarès, France, by car (3‐h journey; 20‐L containers of oxygenated saltwater; ca. 70 fish of average mass 0.02 g per litre). Two separate cohorts were obtained for Experiment 1 (arrival date: 20/01/2014) and Experiment 2 (arrival date: 10/06/2014).

Seabass were kept at the experimental laboratory in plastic, rectangular stock tanks (height: 88 cm; width: 54 cm; length: 66 cm; wall thickness: 3 mm) containing 290 L of filtered saltwater (water height: 80 cm) and a slow‐bubbling airstone. Water temperature was 19 ± 1 °C; lighting was provided 12:12 day:night; filtration was via a closed‐water recirculation system (TMC System 5000P Marine Reservoir‐based Filtration Unit). Fish were fed on commercial aquaculture pellets (Skretting, Norway); initially feeding was multiple times per day to avoid cannibalism; during long‐term experiments, feeding was once per day; all tanks received the same feeding regime throughout.

### Sound recordings and playback tracks

Experimental playback tracks were created using Audacity 1.3.13 (http://audacity.sourceforge.net/) from original field recordings (as per Wale *et al*., 2013a; Simpson *et al*., 2015). Recordings of ambient coastal noise were made at three major UK harbours (Gravesend, Plymouth and Portsmouth) when there were no ships passing close by. Recordings of ship noise were made at the same three harbours when a single ship was passing at ca. 100‐ to 400‐m distance (Gravesend: Rio de la Plata, a 286 m long, 64 730‐t container ship; Plymouth: Bro Distributor, a 147 m long, 14 500‐t LPG tanker; Portsmouth: Commodore Goodwill, a 126 m long, 5215‐t ferry). Ships were travelling at constant, relatively slow speeds (<10 knots), as enforced by port authorities for vessels entering and leaving estuarine areas. Recordings of ambient noise and ship passes were made using a hydrophone (HTI‐96‐MIN with inbuilt preamplifier, High Tech Inc., Gulfport MS; manufacturer‐calibrated sensitivity −164.3 dB re 1 V *μ*Pa^−1^; frequency range 0.2–30 kHz), positioned at 1 m depth 20–40 m offshore, and a digital recorder (Edirol R‐09HR, 44.1 kHz sampling rate, Roland, Hamamatsu, Japan). The recording level was calibrated using pure sine wave signals from a function generator with a measured voltage recorded in line on an oscilloscope.

Recordings of pile‐driving in Swansea Bay, United Kingdom, were made 127 m from the sound source (a 1.2‐m‐diameter monopole driven ca. 25 m into the seabed with a 6.5 m water depth), with a hydrophone (HTI‐99HF, High Tech Inc., Gulfport MS; manufacturer‐calibrated sensitivity −204 dB re 1 V *μ*Pa^−1^; 0.02–125 kHz frequency range) at 2–3 m depth connected to a data logger (RTsys, Caudan, France). Recordings of a seismic array (4450 cubic inches) in the Santos Basin, Brazil, were made 329 m from the sound source (closest distance of a towed array which passed the hydrophone) using a hydrophone (Seiche; manufacturer‐calibrated sensitivity −201 dB re 1 V *μ*Pa^−1^; frequency range 0.01–200 kHz) connected to a digital recorder (RME Fireface 800, 48 kHz sampling rate: Haimhausen, Germany). All recordings were made during still‐to‐moderate wind speeds.

For each of the four sound types (recordings of ambient, ship, pile‐driving and seismic noise), two sets of playback tracks were made: one set (three of each sound type) for use in short‐term experiments and one set (six of each sound type) for use in long‐term experiments. The use of multiple tracks for each sound type and time frame reduced issues of pseudoreplication. Short‐term experimental tracks were all 5 min in duration. For ambient and pile‐driving playbacks, a random part of the relevant recording was used; for ship and seismic playbacks, the chosen 5 min was from the maximum amplitude period of the recording (i.e. when the vessel was closest to the hydrophone).

The composition of playback tracks for the long‐term experimental tanks differed between treatments to reflect the four acoustic scenarios (see Figs S1 and S2). Each ambient‐noise tank was allocated a unique combination of four of six possible 1‐h coastal recordings that played on a continuous shuffled programme. Each ship‐noise tank was allocated a unique combination of four of six possible 1‐h tracks, which each had a single 15‐min ship pass starting at 20 or 40 min (5‐min fade in, 5‐min full amplitude and 5‐min fade out) and ambient noise in between; by randomly shuffling the tracks, ship passes were 25, 45 or 65 min apart to avoid predictability. Each tank with pile‐driving playback was allocated a unique combination of four of six possible 6‐h tracks, with 4 h of ‘constant’ pile‐driving (one strike approx. every 1.5 s with ambient noise between strikes) and 1 h of ambient noise at the start and finish; on a random shuffle, this gave 2 h of ambient noise followed by 4 h of pile‐driving on a continuous cycle. Each seismic‐noise tank was allocated a unique combination of four of six possible 2‐h tracks, which each had 1 h of ‘constant’ airgun noise (a ship approaching and passing, towing a seismic airgun which let off blasts once every 12 s) and 1 h of ambient noise in either order; by randomly shuffling the tracks, seismic survey noise could play for 2 h continuously or have a 1‐ or 2‐h period of ambient noise in between periods of seismic noise.

Playbacks were via underwater loudspeakers (UW‐30; max output level 156 dB re 1 *μ*Pa at 1 m, frequency response 0.1–10 kHz; University Sound, Whitehall, Ohio, USA) resting on a foam base at the bottom of the tank and facing upwards. Recordings of playbacks in stock tanks were made in the centre of the tank and 45 cm above the tank floor, using the same hydrophone as for ambient and ship recordings and a digital recorder (Sony PMC‐M10, 44.1 kHz sampling rate, Sony Corporation, Tokyo, Japan). Due to unresolved challenges in measuring particle motion in small tanks at the time of the experiments, we assessed acoustic conditions in the pressure domain only. In this study, we do not attempt to establish absolute values for sensitivity, but rather explore the potential for animal responses to change as a consequence of repeated exposure to additional noise of different sound types.

### Acoustic analysis

**Figure 1:**
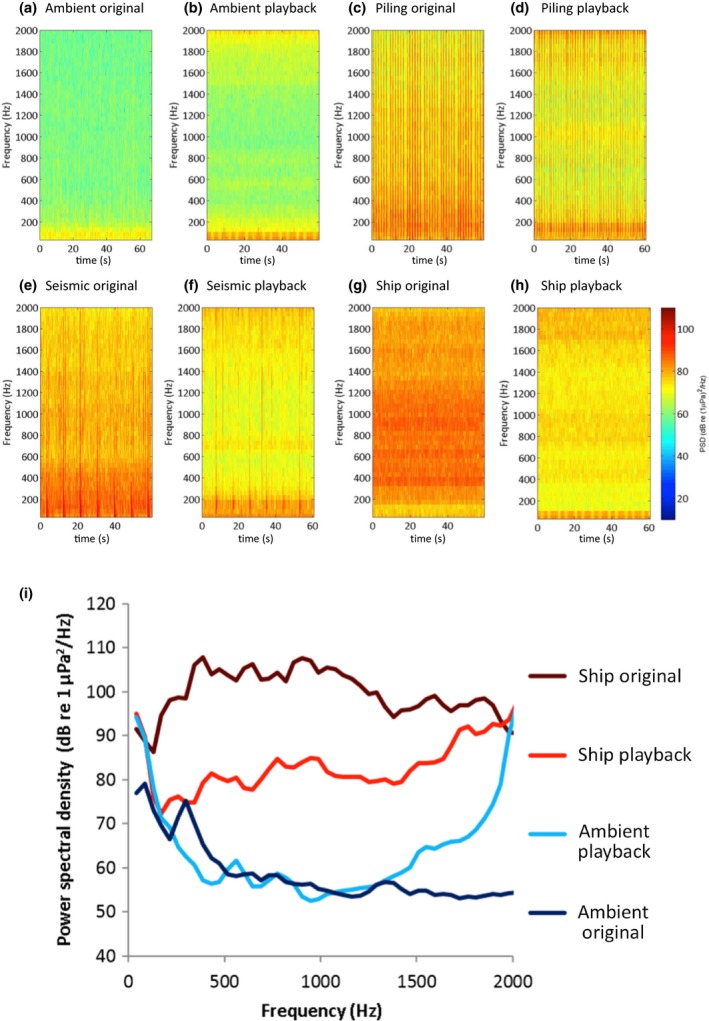
(a–h) Illustrative spectrograms of the four sound types used in the experiments, showing both examples from an original recording and from the recording of playback in one of the long‐term exposure tanks. (i) Power spectral densities of sound pressure levels from recordings of original ambient and ship conditions and playback of those recordings in a long‐term exposure tank. Playbacks were affected by near‐field effects, and speaker performance meaning some frequencies were louder and others quieter, but ships were louder than ambient noise and ship‐noise playbacks were louder than ambient‐noise playbacks. Sounds <10 Hz are unlikely to be generated by the speakers, but may result from, for example, background pump noise or vibrations in the experimental laboratory. The higher levels at >1500 Hz for ambient‐noise playbacks compared to original ambient‐noise recordings likely result from background noise, the resonant frequency of the tank, and the frequency response of the playback set‐up.

Sound recordings were analysed in MATLAB 2013a using the analysis package from Merchant *et al*. (2015). Recordings were low‐pass filtered at 2 kHz prior to analysis to focus on the frequencies of most likely relevance (those below 1 kHz) to seabass hearing (Lovell, 2003). Spectrograms and power spectral densities (see Fig. 1) were calculated using a window length of 1024 over a 1‐min recording. Root‐mean‐squared (RMS) levels and consistency at 130 and 140 dB for all treatments, and peak levels for ambient and ship treatments, were calculated over 1‐min samples. Peak levels, 90% energy envelope, rise time and single‐strike sound‐exposure level (SELss) were averaged over five different randomly selected impulses for pile‐driving and seismic treatments.

### Experimental design

Our focus in this study was the effect of repeated exposure to additional noise; comparisons were made with individuals from the same cohort from the same holding conditions that experienced control playbacks (of recordings of ambient coastal noise) and so any treatment‐based effect is not the consequence of captive conditions *per se*. Individual seabass were tested once in an independent‐samples design; different cohorts were used for the two experimental sets. Both experimental sets constituted three phases (short‐term experiment, long‐term experiment and coupled short‐term experiment). In experimental set 1 (January–April 2014), we compared responses to an impulsive sound type (playback of recordings of pile‐driving noise) with a more continuous sound type (playback of recordings of ship noise); playback of recordings of ambient coastal noise was used as a control. In experimental set 2 (June–September 2014), we compared responses to two different impulsive sound types (playback of recordings of pile‐driving and seismic noise); playback of recordings of ambient coastal noise was again used as a control.

#### Phase 1: Short‐term experiment

To test the immediate effect of a single short‐term exposure to additional noise, we used a physiological measure because changes in behaviour do not always provide a sufficiently sensitive or timely indicator of a response to a stimulus (Beale & Monaghan, 2004). Specifically, we considered ventilation rate (measured as opercular beat rate; OBR). Ventilation rate is a recognized secondary indicator of stress (Barton, 2002), is a robust measure allowing control for the baseline OBR of individual fish in a matched design, is easily measured by an observer who is blind to the acoustic experience of each fish and has previously been shown to be affected by anthropogenic noise (Simpson *et al*., 2015; Bruintjes *et al*., 2016).

Postlarval seabass were tested within 1 week of arrival at the experimental laboratory, having been exposed to no playback tracks previously; they had been kept in stock conditions exposed only to tank noise. For testing, individual seabass were placed into plastic containers (height: 12 cm; width: 13 cm; length: 18.5 cm; wall thickness: 1.5 mm; water volume: 280 ml) inside a glass test tank (height: 32.5 cm; width: 32 cm; length: 63 cm; wall thickness: 3 mm; water volume: 60 L) at a fixed location 30 cm from a sideward‐facing loudspeaker (details above) suspended at one end. Seabass were allowed to settle for 2 min while an ambient track was playing. An observer then counted opercular beats for 1 min. If opercular beats could not be observed, counting was paused; for every individual tested, a full 1 min of beats was counted (always within 90 s). There was then a switch to the designated experimental track (one of the three sound types, including ambient, for that experimental set), and 1 min of opercular beats was counted as before. Time was monitored and the track was switched by a second observer.

The tubes were cleaned and the water replaced with fully aerated saltwater after each seabass (to prevent any accumulation of stress hormones), and we tested fish in five blocks of 18 individuals in each experimental set. Within each block, equal numbers of fish received the three experimental sound types, with order randomly allocated within each block; subsequent analysis confirmed that this did not result in any chance bias in the ordering of different sound treatments (Kruskal–Wallis tests on ranked orders: all *P *>* *0.118). Following OBR counting, all tested fish were weighed using a G&G GmbH pocket scale (Neuss, Germany) and measured (standard length; 15 cm metal ruler).

#### Phase 2: Long‐term experiment

One hundred and fifty postlarval seabass were placed in each of nine stock tanks for each experimental set. The three sound treatments in a given experimental set were assigned to three stock tanks each; tanks contained an upward facing loudspeaker (details above). Fish were kept in the stock tanks for 12 weeks, throughout which the relevant noise was played on a continuous randomized cycle (see Sound recordings and playback tracks). Feeding, water temperature, lighting conditions and recirculation were as per general husbandry (see Holding conditions). Each week, 40 fish were temporarily removed from each tank for weighing (30 fish in three groups of 10; Ohaus Valor 300 series scale, Parsippany, USA) and measuring (10 fish individually for standard length; 15 cm ruler); fish were immediately replaced in their stock tank afterwards. Each week, the number of deaths per tank was also recorded; dead fish were removed daily.

#### Phase 3: Coupled short‐term experiment

At the end of the 12‐week sound exposure, subsets of fish from each tank were tested for their response to short‐term exposure to one of the different sound treatments in that experimental set using ventilation rate as the response measure (same general methods as for the short‐term experiment). For each fish, the initial playback period (counting of baseline OBR) was of their home‐tank track, with a switch to a different track from one of the three sound types for the second period of OBR counting. Thirty fish from each of the nine tanks were tested; 10 each with one of the three sound types as the experimental track. Fish were tested in 10 blocks of 27 fish (one each of fish from every stock tank and all three sound types) in each experimental set. The order of testing within blocks was randomized; subsequent analysis confirmed that this did not result in any chance bias in the ordering of different sound treatments (Kruskal–Wallis tests on ranked orders: all *P *>* *0.740). Following OBR counting, all tested fish were weighed and measured (as in the short‐term experiment).

### Statistical analysis

All data were analysed using SPSS version 21 (IBM Corp., Armonk, NY, USA). For all tests, normality of residuals and heteroscedasticity of variances was checked and parametric tests (on raw or transformed data) or nonparametric tests conducted as appropriate (details below). In all analyses, interactions between fixed terms were checked but never found to be significant and so are not presented in the Results.

To analyse OBR data from the short‐term experiments, general linear models (GLMs) were used, with the change in OBR from initial ambient playback period to experimental playback period included as the response measure. We controlled for testing block and fish size (model outputs are presented throughout the Results using length measurements, but qualitatively the same findings were apparent if mass was used), while examining the effect of experimental sound treatment (experimental set 1: ambient, ship, pile‐driving; experimental set 2: ambient, pile‐driving, seismic).

To analyse all other data sets, we used mixed models to control for the testing of multiple fish from the same stock tanks, which are not therefore independent. For the long‐term experimental data, we controlled for fixed effects of testing block and fish size, along with random effect of tank identity, while examining the effect of sound treatment. In the case of fish growth, we ran separate linear mixed models (LMMs) for mass (square‐root‐transformed) and length. We ran generalized linear mixed models (GLMMs) with a Poisson distribution and a logit link function to consider weekly counts of dead fish.

We also used mixed models to consider data from the coupled short‐term experiments, examining how fish that had been exposed to 12 weeks of a given sound treatment responded to a short‐term exposure to that sound or a different sound type. To determine the baseline OBR of fish from different rearing conditions, the OBR in the initial playback period (home‐tank noise) was used as the response variable. The change in OBR from initial playback period to experimental playback period was used as the response variable in other analyses. In each case, we controlled for the fixed effects of testing block and fish size (as above), as well as the random effect of home‐tank identity.

## Results

### Acoustics

Ambient playbacks had the lowest RMS level and consistency at 130 dB, followed by ship, seismic and pile‐driving playbacks, respectively (Table 1). Impulsive pile‐driving playbacks had a 90% energy envelope 72 times shorter and rise time two times shorter than impulsive seismic playbacks (Table 1). The peak levels and SELss of pile‐driving playbacks were 4–5 dB higher than seismic playbacks (Table 1). Playbacks differed to original recordings because of the frequency response of the loudspeakers used, near‐field effects and interference due to the unavoidable reflections and reverberations within tanks (see Fig. 1 for a comparison of the power spectral densities of original and played‐back ambient and ship noise).

**Table 1:** Acoustic comparisons of playback tracks used in long‐term experiments. Sound recordings were analysed in MATLAB 2013a using the paPAM analysis package (Merchant *et al*., 2015); full details provided in main text

Noise playback	RMS level (60s) (dB re 1 *μ*Pa)	Consistency at 130 dB	Consistency at 140 dB	Peak level (dB re 1 *μ*Pa)	90% energy envelope (ms)	Rise time (ms)	SELss (dB re 1 *μ*Pa^2^*s)
Ambient	117.23	0.65	0.00	141.20	NA	NA	NA
Ship	124.71	6.53	0.00	138.63	NA	NA	NA
Pile‐driving	146.66	25.49	7.72	163.31	142.65	39.10	147.40
Seismic	131.54	11.91	0.28	158.39	10285.30	77.51	143.48

### Experimental set 1

**Figure 2:**
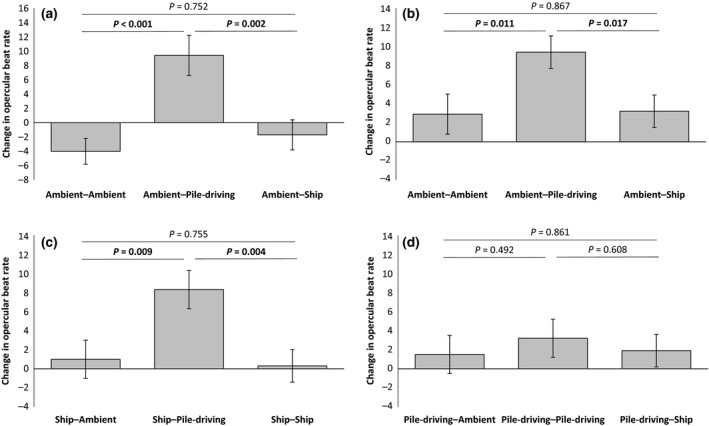
Change in opercular beat rate of seabass in experimental set 1 during two consecutive short‐term (2 min) exposures to playback of recordings of different sounds (ambient noise, pile‐driving noise or ship noise). In (a) are responses of ‘naïve’ (no prior experience of playbacks) postlarval individuals to ambient‐noise playback followed by playback of one of the three sounds (*n* = 90 evenly spread between the three treatments). In (b–d) are responses of individuals that have experienced 12 weeks exposure to ambient‐noise playback, pile‐driving‐noise playback or ship‐noise playback, respectively; testing involved a change from playback of the long‐term noise exposure to a different playback track (*n* = 90 evenly spread between treatments in each case). Shown in all cases are means ± SE, with the significance of pairwise *post hoc* tests indicated above bars (significant results in bold).

Sound treatment had a significant effect on the OBR of naïve postlarval seabass (GLM: *F*_2,82_ = 8.85, *P* < 0.001; Table S1). Short‐term exposure to pile‐driving noise resulted in a significantly greater increase in OBR than short‐term exposure to either ambient noise or ship noise; there was no significant difference in the OBR change exhibited by fish exposed short‐term to ambient or ship noise (Fig. 2a).

Following 12 weeks of exposure to ambient noise, seabass still exhibited the same significant difference in response to the short‐term sound treatments (LMM: *F*_2,70.2_ = 4.22, *P* = 0.019; Table S2a): fish reared in ambient noise exhibited a significantly greater increase in OBR when exposed in the coupled short‐term experiment to pile‐driving noise compared to either ambient noise or ship noise; there was no significant difference in the OBR change exhibited by ambient‐reared fish exposed short‐term to ambient or ship noise (Fig. 2b). Qualitatively similar results were obtained for seabass reared in ship noise, with the coupled short‐term sound treatment having a significant effect on OBR change (*F*_2,73_ = 5.39, *P* = 0.007; Table S2b): fish reared in ship noise showed a significantly greater increase in OBR in response to short‐term pile‐driving noise compared to either ambient noise or ship noise; there was no significant difference in the OBR change exhibited by ship‐reared fish exposed short‐term to ambient or ship noise (Fig. 2c). However, a different result was found for seabass reared in pile‐driving noise as these individuals exhibited no significant difference in response to subsequent short‐term exposure to different sound treatments (*F*_2,74.9_ = 0.26, *P* = 0.773; Table S2c). For these fish, short‐term pile‐driving noise did not result in a significantly different change in OBR compared to short‐term ambient or ship noise (Fig. 2d).

Fish from the three long‐term sound‐exposure treatments did not differ significantly in their baseline OBR (LMM: *F*_2,234_ = 0.29, *P* = 0.761; Table S3a). Nor was there any significant difference in the growth rates (length: *F*_2,1070_ = 0.67, *P* = 0.544; Table S3b; mass: *F*_2,314_ = 0.30, *P* = 0.752; Table S3c) or mortality rate (GLMM: *F*_2,92_ = 1.21, *P* = 0.228; Table S3d) of fish in the three long‐term sound‐exposure treatments.

### Experimental set 2

**Figure 3:**
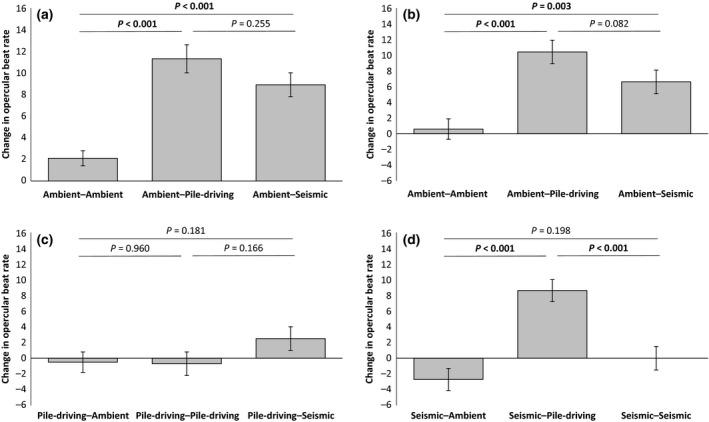
Change in opercular beat rate of seabass in experimental set 2 during two consecutive short‐term (2 min) exposures to playback of recordings of different sounds (ambient noise, pile‐driving noise or seismic noise). In (a) are responses of ‘naïve’ (no prior experience of playbacks) postlarval individuals to ambient‐noise playback followed by playback of one of the three sounds (*n* = 90 evenly spread between the three treatments). In (b–d) are responses of individuals that have experienced 12 weeks exposure to ambient‐noise playback, pile‐driving‐noise playback or seismic‐noise playback, respectively; testing involved a change from playback of the long‐term noise exposure to a different playback track (*n* = 90 evenly spread between treatments in each case). Shown in all cases are means ± SE, with the significance of pairwise *post hoc* tests indicated above bars (significant results in bold).

Sound treatment had a significant effect on the OBR of naïve postlarval seabass (GLM: *F*_2,82_ = 20.37, *P* < 0.001; Table S4). Short‐term exposure to both pile‐driving and seismic noise resulted in a significantly greater increase in OBR than short‐term exposure to ambient noise; there was no significant difference in the OBR change exhibited by fish exposed short‐term to pile‐driving and seismic noise (Fig. 3a).

Following 12 weeks of exposure to ambient noise, seabass still exhibited the same significant difference in response to the short‐term sound treatments (LMM: *F*_2,77_ = 12.10, *P* < 0.001; Table S5a): fish reared in ambient noise exhibited a significantly greater increase in OBR when exposed in the coupled short‐term experiment to either pile‐driving or seismic noise compared to ambient noise; there was a strong, but statistically nonsignificant trend for a greater increase in OBR in response to short‐term pile‐driving compared to seismic noise (Fig. 3b). Seabass exposed to 12 weeks of seismic noise also exhibited a significant difference in OBR response depending on sound treatment in the coupled short‐term experiment (*F*_2,77_ = 16.44, *P* < 0.001; Table S5b). However, the difference here was that seismic‐reared fish did not exhibit a significant difference in OBR change when exposed to either short‐term ambient or seismic noise, but still exhibited a significantly greater increase in OBR when experiencing short‐term exposure to pile‐driving noise (Fig. 3c). Seabass exposed to 12 weeks of pile‐driving noise showed no significant difference in OBR response to the three sound treatments in the coupled short‐term experiment (LMM: *F*_2,77_ = 1.26, *P* = 0.290; Table S5c). That is, these fish not only showed no significantly greater increase in OBR in response to short‐term pile‐driving noise compared to short‐term ambient noise, but also exhibited no significantly different response to short‐term seismic noise compared to ambient noise (Fig. 3d).

Fish from the three long‐term sound‐exposure conditions did not differ significantly in their baseline OBR (LMM: *F*_2,251.0_ = 1.32, *P* = 0.337; Table S6a). Nor was there any significant difference in the growth rates (length: *F*_2,1160_ = 0.39, *P* = 0.691; Table S6b; mass: *F*_2,341_ = 0.21, *P* = 0.979; Table S6c) or mortality rate (GLMM: *F*_2,101_ = 0.89, *P* = 0.371; Table S6d) of fish in the three long‐term sound‐exposure conditions.

## Discussion

Naïve seabass exposed to impulsive sounds (playbacks of recordings of pile‐driving and seismic surveys), but not a more continuous sound type (playback of recordings of ship noise), responded with an elevated OBR relative to control individuals exposed to ambient‐noise playback. An increased ventilation rate in response to additional noise (see also Simpson *et al*., 2015; Bruintjes *et al*., 2016) is indicative of increased stress (Barton, 2002). However, rearing in impulsive‐noise conditions for 12 weeks resulted in a lessened OBR response to additional noise; fish reared with seismic‐noise playback exhibited a reduced response just to that sound type, but fish reared with playback of pile‐driving noise exhibited a reduced response to both pile‐driving and seismic‐noise playbacks. This is strong experimental evidence that the response to noise can change with repeated exposure. Given this lessened response, it is perhaps not surprising that fish reared in different sound treatments did not differ in their baseline stress levels (as indicated by ventilation rate), growth at 12 weeks or mortality. These findings demonstrate why caution is needed when drawing conclusions about fitness consequences from single short‐term experiments (see also Bejder *et al*., 2006). Such conclusions may be accurate if considering responses with immediate fitness outcomes, such as antipredator behaviour (see Wale *et al*., 2013b; Simpson *et al*., 2015, 2016), but are not necessarily so if there is a chance for animals to compensate over time.

The documented lessening of response to impulsive noise could theoretically arise from mortality of the most susceptible individuals, leaving only those with high initial tolerance for testing at the end of the exposure period. Intrapopulation variation in vulnerability to noise is certainly expected with respect to, for example, sex, age, size and condition (Wale *et al*., 2013a; Radford *et al*., 2015), but mortality rates in the current experiments were generally low (mean: 10% in 12 weeks) and deaths in all sound treatments were similar. In our tank‐based set‐up, there was also no possibility for less tolerant individuals to move away; there was no likelihood that our comparison at the start and end of the noise‐exposure period was of different cohorts of individuals (cf., e.g. Thompson *et al*., 2013). Nor can changes in response be the indirect consequences of noise effects on other species with which the focal animals interact (see Bejder *et al*., 2009) because seabass were reared alone in the experimental tanks. There remain, therefore, two potential explanations for the reduced response with repeated impulsive‐noise exposure: a change in tolerance or a shift in hearing threshold.

Increased tolerance can arise from habituation, a learned reduction in response to a stimulus as organisms come to realize that it does not have detrimental consequences (Bejder *et al*., 2009). Development of increased tolerance has previously been shown in other contexts (Ellenberg *et al*., 2009; Ensminger & Westneat, 2012; Viblanc *et al*., 2012), but rarely considered with respect to anthropogenic noise (see Nedelec *et al*., 2015, in press). Such a lessening of response has implications for the projected impacts of anthropogenic noise. It has often been suggested in studies looking at single short‐term noise exposures that there could be lasting consequences of the effects seen. But, if increased tolerance can develop, and if it can do so relatively quickly, then there may be a reduced likelihood of negative fitness consequences (see also Bejder *et al*., 2006). Certainly, we found no evidence for any effect on mortality or growth after 12 weeks of exposure, even for the impulsive sounds that had the largest short‐term impact. The lack of an effect on growth after a few weeks of exposure is in line with most previous work exploring the impacts of anthropogenic noise on fish (Wysocki *et al*., 2007; Bruintjes & Radford, 2014; Nedelec *et al*., 2015, in press; but see Anderson *et al*., 2011). If growth had been affected earlier on (see Davidson *et al*., 2009; Nedelec *et al*., 2015), catch‐up growth can be detrimental to fitness due to oxidative stress (Lee *et al*., 2013), but there appeared to be no treatment‐based effects on growth at any stage in the experimental exposure period. However, there could have been other effects that we did not measure, such as on telomere length (see Meillère *et al*., 2015).

Previous work on fish hearing has shown evidence for a noise‐induced temporary threshold shift (TTS) in some species (Scholik & Yan, 2001; Popper *et al*., 2005; Wysocki *et al*., 2007). Further studies to determine the hearing thresholds of seabass at low frequencies (cf. Lovell, 2003) and to assess whether the sound levels in the current experiment could induce TTS in the study species are needed. However, if TTS is the explanation for the demonstrated reduction in response to impulsive sound types following long‐term exposure, then the implications differ somewhat compared to if an increased tolerance is the underpinning mechanism. In both cases, any initial increases in stress or distraction caused by additional noise are likely to be lessened over time (see above). But, TTS could have the knock‐on consequences of a reduced responsiveness to other, useful, sounds such as the acoustic cues and signals cues used by many fishes for orientation and settlement, detection of predators and prey, and for communication (Popper *et al*., 2003; Radford *et al*., 2014).

The acoustic properties of impulsive playbacks may affect the development and generalization of a reduced response, because exposure to playbacks of recordings of seismic surveys resulted in a lessened impact of just that sound type, but exposure to playbacks of recordings of pile‐driving led to a reduced response to both that sound type and of seismic‐noise playbacks. RMS level, consistency at 130 dB, peak level and number of exposures per minute were all higher for pile‐driving than seismic playbacks. Rise time and 90% energy envelopes also differed between the two impulsive experimental sounds, being shorter for pile‐driving than seismic playbacks. These acoustic properties may have meant that pile‐driving playbacks were more startling or aversive, or more likely to generate a TTS, than seismic playbacks (Gotz & Janik, 2010). The frequency content of impulsive playbacks may also have affected responses to them; it is possible that pile‐driving playbacks were louder at frequencies that were in the range of best hearing in the seabass than seismic playbacks, meaning an increased perceived loudness of pile‐driving playback. Increased tolerance or a greater hearing threshold shift to the more startling or aversive sound stimulus (pile‐driving playback) may have resulted in the generalization of reduced responsiveness to include the less startling or aversive sound stimulus (seismic playback).

Tank‐based playback experiments allow valuable assessment of principles relating to the impact of sound stimuli, variation in responses dependent on differing acoustic properties and the potential for changes in responses (Radford *et al*., 2015; Slabbekoorn, 2015). Recent work has also demonstrated qualitatively similar findings from experiments involving the exposure of fish to playbacks of anthropogenic noise in tanks and experiments involving the exposure of fish in open‐water conditions to real anthropogenic‐noise sources (Simpson *et al*., 2016). However, it is important to remember that there are both behavioural and acoustic limitations to tank‐based playback experiments, including that the speakers do not generate sound in the lowest frequency ranges, that experiments are conducted in the near field and that the sound field, especially in the particle motion domain, will differ compared to that in open‐water conditions (Rogers, 2015; Slabbekoorn, 2015). In our experiments, the ambient‐noise (control) treatment was also relatively loud (mean RMS level (60s) = 117.23 dB re 1 *μ*Pa; Table 1), in comparison with measurements of real ocean noise (e.g. Andrew *et al*., 2011). This is likely due to noise from, for example, the pumps required to keep fish alive during the 12‐week exposure period, and hence also explains the louder conditions compared to previous laboratory‐based, short‐term exposure experiments conducted in tanks without pumps (e.g. Simpson *et al*., 2015). However, since we still find a significant effect of the impulsive sound types (playback of recordings of pile‐driving and seismic noise) compared to playback of ambient‐noise recordings, and since fish exposed long term to these control conditions still exhibited the same responses as ‘naïve’ fish to short‐term exposure to the impulsive sound types, we believe our results are conservative; an even larger difference might have been expected if the control conditions were quieter.

If absolute measures of the impact of particular noises or dose‐dependent responses are required for management decisions by regulators, then experiments in natural conditions with real‐world noise sources are required. Those are much more logistically challenging (but see Debusschere *et al*., 2016), especially with respect to controlled long‐term exposure experiments as presented here. Future work also needs to tease apart potential underpinning mechanisms for a change in response; in the case of the reduction in response documented here, that would mean examining which of TTS or increased tolerance plays the key role. For now, the current work provides strong empirical evidence of the need for repeated‐ or chronic‐exposure experiments because short‐term experiments do not necessarily provide a complete picture of responses and do not reflect most anthropogenic‐noise scenarios in the natural world.

## Supporting information


**Figure S1.** Example 6‐h programmes of three acoustic treatments in each of the nine tanks during long‐term experimental playback in Experimental Set 1.
**Figure S2.** Example 6‐h programmes of three acoustic treatments in each of the nine tanks during long‐term experimental playback in Experimental Set 2.
**Table S1.** Experimental Set 1 GLM examining how short‐term exposure to three sound treatments (ambient‐noise playback, ship‐noise playback and pile‐driving‐noise playback) affect the change in ventilation rate of ‘naïve’ post‐larval seabass (*n* = 90).
**Table S2.** Experimental Set 1 LMMs examining how the ventilation rate of juvenile seabass reared in three different long‐term (12 week) noise‐exposure conditions – (a) ambient‐noise playback, (b) ship‐noise playback, (c) pile‐driving‐noise playback – is affected by short‐term exposure to playback of one of the same three noise treatments (*n* = 90 in each long‐term cohort).
**Table S3.** Experimental Set 1 mixed models examining how long‐term (12 week) exposure to one of three sound treatments (ambient‐noise playback, ship‐noise playback, pile‐driving‐noise playback) influences juvenile seabass (a) baseline ventilation rate (LMM; *n* = 270 fish), (b) length (LMM; 1080 measurements), (c) mass (LMM; 324 measurements), and (d) mortality (GLMM; 99 weekly counts).
**Table S4.** Experimental Set 2 GLM examining how short‐term exposure to three sound treatments (ambient‐noise playback, seismic‐noise playback and pile‐driving‐noise playback) affect the change in ventilation rate of ‘naïve’ post‐larval seabass (*n* = 90).
**Table S5.** Experimental Set 2 LMMs examining how the ventilation rate of juvenile seabass reared in three different long‐term (12 week) noise‐exposure conditions – (a) ambient‐noise playback, (b) seismic‐noise playback, (c) pile‐driving‐noise playback – is affected by short‐term exposure to playback of one of the same three noise treatments (*n* = 90 in each long‐term cohort).
**Table S6.** Experimental Set 2 mixed models examining how long‐term (12 week) exposure to one of three sound treatments (ambient‐noise playback, seismic‐noise playback, pile‐driving‐noise playback) influences juvenile seabass (a) baseline ventilation rate (LMM; *n* = 270 fish), (b) length (LMM; 1170 measurements), (c) mass (LMM; 351 measurements), and (d) mortality (GLMM; 108 weekly counts).
