# Supplementary material for: Repeated exposure reduces the response to impulsive noise in European seabass
Source: Glob Chang Biol. 2016 Jun 10;22(10):3349–60. doi: 10.1111/gcb.13352 (PMC5006868; doi:10.1111/gcb.13352)
Supplement: Supplementary file 1 — Figure S1. Example 6‐h programmes of three acoustic treatments in each of the nine tanks during long‐term experimental playback in Experimental Set 1. Figure S2. Example 6‐h programmes of three acoustic treatments in each of the nine tanks during long‐term experimental playback in Experimental Set 2. Table S1. Experimental Set 1 GLM examining how short‐term exposure to three sound treatments (ambient‐noise playback, ship‐noise playback and pile‐driving‐noise playback) affect the change in ventilation rate of ‘naïve’ post‐larval seabass (n = 90). Table S2. Experimental Set 1 LMMs examining how the ventilation rate of juvenile seabass reared in three different long‐term (12 week) noise‐exposure conditions – (a) ambient‐noise playback, (b) ship‐noise playback, (c) pile‐driving‐noise playback – is affected by short‐term exposure to playback of one of the same three noise treatments (n = 90 in each long‐term cohort). Table S3. Experimental Set 1 mixed models examining how long‐term (12 week) exposure to one of three sound treatments (ambient‐noise playback, ship‐noise playback, pile‐driving‐noise playback) influences juvenile seabass (a) baseline ventilation rate (LMM; n = 270 fish), (b) length (LMM; 1080 measurements), (c) mass (LMM; 324 measurements), and (d) mortality (GLMM; 99 weekly counts). Table S4. Experimental Set 2 GLM examining how short‐term exposure to three sound treatments (ambient‐noise playback, seismic‐noise playback and pile‐driving‐noise playback) affect the change in ventilation rate of ‘naïve’ post‐larval seabass (n = 90). Table S5. Experimental Set 2 LMMs examining how the ventilation rate of juvenile seabass reared in three different long‐term (12 week) noise‐exposure conditions – (a) ambient‐noise playback, (b) seismic‐noise playback, (c) pile‐driving‐noise playback – is affected by short‐term exposure to playback of one of the same three noise treatments (n = 90 in each long‐term cohort). Table S6. Experimental Set 2 mixed models examining how l [file GCB-22-3349-s001.docx]

**Repeated exposure reduces the response to impulsive noise
in European seabass**

**Andrew N. Radford, Laurie Lèbre, Gilles Lecaillon, Sophie L. Nedelec & Stephen D. Simpson**

**Supplementary Material**

Details of the composition of playback tracks for the four sound treatments in the long-term experiments (referred to in Methods of main paper) are provided in Supplementary Figures S1 & S2.

Full output from statistical models (referred to in Results of main paper) are provided in Supplementary Tables S1–S6.

**Supplementary FigureS1.** Example 6 h programmes of three acoustic treatments in each of the nine tanks during long-term experimental playback inExperimental Set 1. Three tanks received each treatment. Each tank received ambient coastal noise from two of the three harbours, with ship and pile-driving noise layered onto the recordings. Ship passes occurred once per hour (15 min duration centred on 20 or 40 min), and pile-driving was for a 4-h period with 2 h breaks. Unique combinations of four of six potential tracks were allocated to each tank, and tracks were played on a random shuffle to avoid predictability.

**Supplementary FigureS2.** Example 6 h programmes of three acoustic treatments in each of the nine tanks during long-term experimental playback inExperimental Set 2. Three tanks received each treatment. Each tank received ambient coastal noise from two of the three harbours, with seismic and pile-driving noise layered onto the recordings. Seismic surveys occurred for 1 h of each 2-h period (first or second hour), and pile-driving was for a 4-h period with 2 h breaks. Unique combinations of four of six potential tracks were allocated to each tank, and tracks were played on a random shuffle to avoid predictability.

**Supplementary Table S1.** Experimental Set 1 GLM examining how short-term exposure to three sound treatments (ambient-noise playback, ship-noise playback and pile-driving-noise playback) affect the change in ventilation rate of ‘naïve’ post-larval seabass (n=90). Parameter estimates provided for significant terms in model; treatment estimates provided relative to ambient-noise treatment as the reference.

| **Predictor** | **F value** | **df** | **P value** | **Parameter estimate** | | |
| --- | --- | --- | --- | --- | --- | --- |
|  |  |  |  | **B** | **SE** | **95% CI** |
| Testing block  Fish length  Sound treatment   - Ambient (control) - Ship - Pile-driving | 0.26  0.02  8.85 | 4,82  1,82  2,82 | 0.904  0.890  <0.001 | 0  2.35  13.28 | -  3.29  3.33 | -  -4.19–8.88  6.66–19.90 |

**Supplementary Table S2.** Experimental Set 1 LMMs examining how the ventilation rate of juvenile seabass reared in three different long-term (12 week) noise-exposure conditions – (a) ambient-noise playback, (b) ship-noise playback, (c) pile-driving-noise playback – is affected by short-term exposure to playback of one of the same three noise treatments (n=90 in each long-term cohort). Parameter estimates provided for significant terms in model; treatment estimates provided relative to ambient-noise treatment as the reference.

| **Predictor** | **F value** | **df** | **P value** | **Parameter estimate** | | |
| --- | --- | --- | --- | --- | --- | --- |
|  |  |  |  | **Estimate** | **SE** | **95% CI** |
| **(a)**  Testing block  Fish length  Sound treatment   - Ambient (control) - Ship - Pile-driving   Tank (random term) | 1.01  0.17  4.22 | 9  1  2 | 0.444  0.682  0.019 | 0  0.44  6.93  3.52 | -  2.60  2.65  7.10 | -  -4.74–5.61  1.65–12.20 |
| **(b)**  Testing block  Fish length  Sound treatment   - Ambient (control) - Ship - Pile-driving   Tank (random term) | 0.97  0.06  5.39 | 9  1  2 | 0.473  0.806  0.007 | 0  -0.84  7.39  0.00 | -  2.69  2.76  0.00 | -  -6.21–4.53  1.90–12.88 |
| **(c)**  Testing block  Fish length  Sound treatment  Tank (random term) | 0.61  0.01  0.26 | 9  1  2 | 0.785  0.907  0.773 | 11.84 | 15.56 |  |

**Supplementary Table S3.** Experimental Set 1 mixed models examining how long-term (12 week) exposure to one of three sound treatments (ambient-noise playback, ship-noise playback, pile-driving-noise playback) influences juvenile seabass (a) baseline ventilation rate (LMM; n=270 fish), (b) length (LMM; 1080 measurements), (c) mass (LMM; 324 measurements), and (d) mortality (GLMM; 99 weekly counts). Parameter estimates provided for significant terms in model.

| **Predictor** | **F value** | **df** | **P value** | **Parameter estimate** | | |
| --- | --- | --- | --- | --- | --- | --- |
|  |  |  |  | **Estimate** | **SE** | **95% CI** |
| **(a)**  Testing block  Fish length  Sound treatment  Tank (random term) | 2.45  0.37  0.29 | 9  1  2 | 0.010  0.541  0.761 | 6.69 | 7.31 |  |
| **(b)**  Week  Sound treatment  Tank (random term) | 7794.75  0.67 | 1  2 | <0.001  0.544 | 2.73  0.20 | 0.03  0.17 | 2.67–2.79 |
| **(c)**  Week  Sound treatment  Tank (random term) | 4988.48  0.30 | 1  2 | <0.001  0.752 | 0.20  0.01 | 0.01  0.01 | 0.20–0.21 |
| **(d)**  Week  Sound treatment  Tank (random term) | 3.15  1.21 | 1  2 | 0.002  0.228 | -0.09  0.02 | 0.03  0.04 | -0.14–-0.03 |

**Supplementary Table S4.** Experimental Set 2 GLM examining how short-term exposure to three sound treatments (ambient-noise playback, seismic-noise playback and pile-driving-noise playback) affect the change in ventilation rate of ‘naïve’ post-larval seabass (n=90). Parameter estimates provided for significant terms in model; treatment estimates provided relative to ambient-noise treatment as the reference.

| **Predictor** | **F value** | **df** | **P value** | **Parameter estimate** | | |
| --- | --- | --- | --- | --- | --- | --- |
|  |  |  |  | **B** | **SE** | **95% CI** |
| Testing block  Fish length  Sound treatment   - Ambient (control) - Seismic - Pile-driving | 1.61  0.18  20.37 | 4,82  1,82  2,82 | 0.180  0.675  <0.001 | 0  6.80  9.07 | -  1.48  1.48 | -  3.86–9.74  6.12–12.02 |

**Supplementary Table S5.** Experimental Set 2 LMMs examining how the ventilation rate of juvenile seabass reared in three different long-term (12 week) noise-exposure conditions – (a) ambient-noise playback, (b) seismic-noise playback, (c) pile-driving-noise playback – is affected by short-term exposure to playback of one of the same three noise treatments (n=90 in each long-term cohort). Parameter estimates provided for significant terms in model; treatment estimates provided relative to ambient-noise treatment as the reference.

| **Predictor** | **F value** | **df** | **P value** | **Parameter estimate** | | |
| --- | --- | --- | --- | --- | --- | --- |
|  |  |  |  | **Estimate** | **SE** | **95% CI** |
| **(a)**  Testing block  Fish length  Sound treatment   - Ambient (control) - Seismic - Pile-driving   Tank (random term) | 1.65  1.32  12.10 | 9  1  2 | 0.117  0.254  <0.001 | 0  6.07  9.59  0.00 | -  1.97  1.98  0.00 | -  2.15–10.01  5.65–13.52 |
| **(b)**  Testing block  Fish length  Sound treatment   - Ambient (control) - Seismic - Pile-driving   Tank (random term) | 1.40  2.24  16.44 | 9  1  2 | 0.201  0.138  <0.001 | 0  2.56  10.91  0.00 | -  1.97  1.99  0.00 | -  -1.37–6.48  6.95–14.87 |
| **(c)**  Testing block  Fish length  Sound treatment  Tank (random term) | 0.40  1.81  1.26 | 9  1  2 | 0.933  0.182  0.290 | 0.00 | 0.00 |  |

**Supplementary Table S6.** Experimental Set 2 mixed models examining how long-term (12 week) exposure to one of three sound treatments (ambient-noise playback, seismic-noise playback, pile-driving-noise playback) influences juvenile seabass (a) baseline ventilation rate (LMM; n=270 fish), (b) length (LMM; 1170 measurements), (c) mass (LMM; 351 measurements), and (d) mortality (GLMM; 108 weekly counts). Parameter estimates provided for significant terms in model.

| **Predictor** | **F value** | **df** | **P value** | **Parameter estimate** | | |
| --- | --- | --- | --- | --- | --- | --- |
|  |  |  |  | **Estimate** | **SE** | **95% CI** |
| **(a)**  Testing block  Fish length  Sound treatment  Tank (random term) | 19.53  1.30  1.32 | 9  1  2 | <0.001  0.255  0.337 | 4.84 | 4.37 |  |
| **(b)**  Week  Sound treatment  Tank (random term) | 8326.81  0.39 | 1  2 | <0.001  0.691 | 2.49  0.71 | 0.03  0.46 | 2.43–2.54 |
| **(c)**  Week  Sound treatment  Tank (random term) | 10658.64  0.02 | 1  2 | <0.001  0.979 | 0.19  0.01 | 0.01  0.01 | 0.19–0.20 |
| **(d)**  Week  Sound treatment  Tank (random term) | 5.80  0.89 | 1  2 | <0.001  0.371 | -0.12  0.06 | 0.02  0.05 | -0.16–-0.08 |
